# Supplementary material for: Immune Monitoring during Therapy Reveals Activitory and Regulatory Immune Responses in High-Risk Neuroblastoma
Source: Cancers (Basel). 2021 Apr 26;13(9):2096. doi: 10.3390/cancers13092096 (PMC8123570; doi:10.3390/cancers13092096)
Supplement: Supplementary file 1 [file cancers-13-02096-s001.zip › cancers-1178913-supplementary.pdf]

cancers

MDPI

Supplementary

Immune Monitoring During Therapy Reveals Activitory and Regulatory Immune Responses in High-Risk Neuroblastoma

Celina L. Szanto et al.

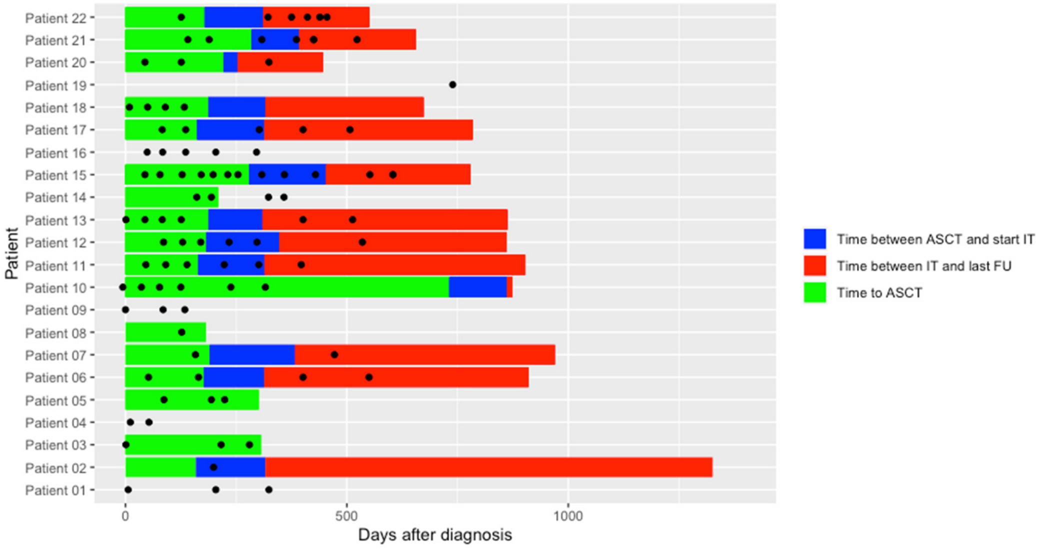

Figure S1. Schematic overview of sampling time points during the HR-NBL treatment course. Patient 4, 9, 16, and 19 did not receive ASCT as a result of disease progression and mortality.

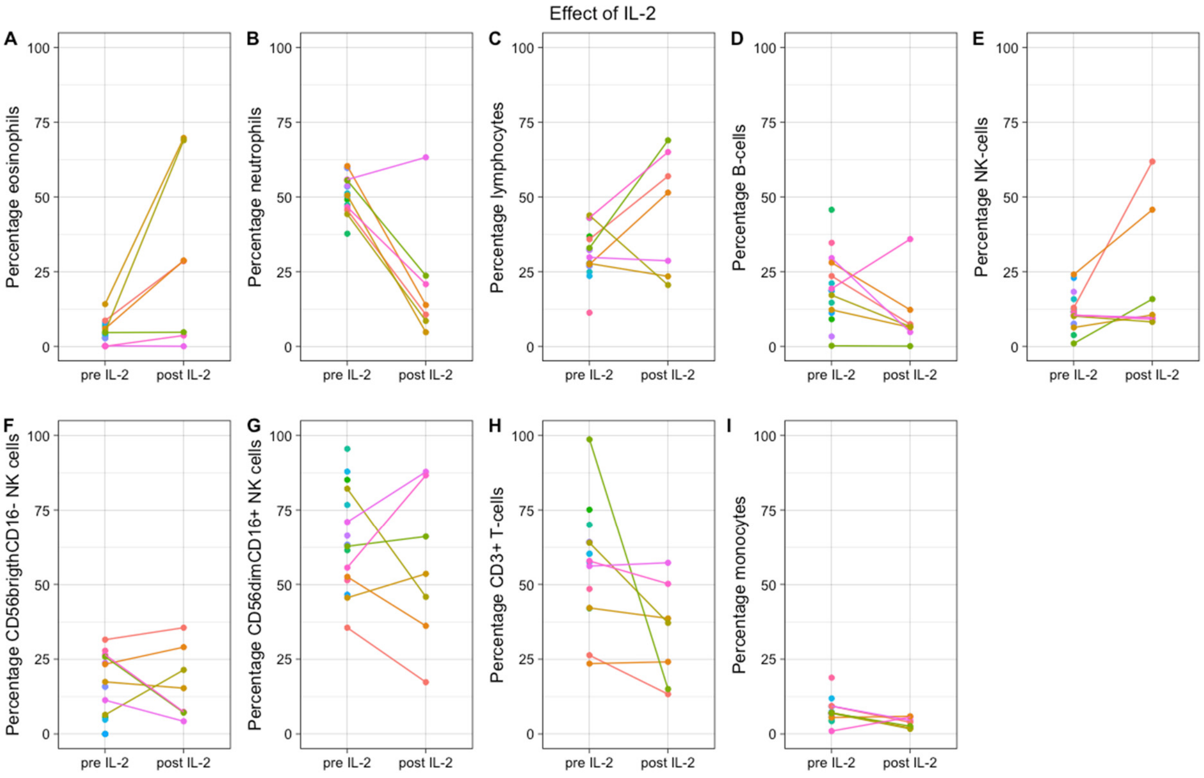

Figure S2. Percentages of cell types based on trucount data before and after IL-2 containing immunotherapy cycles.

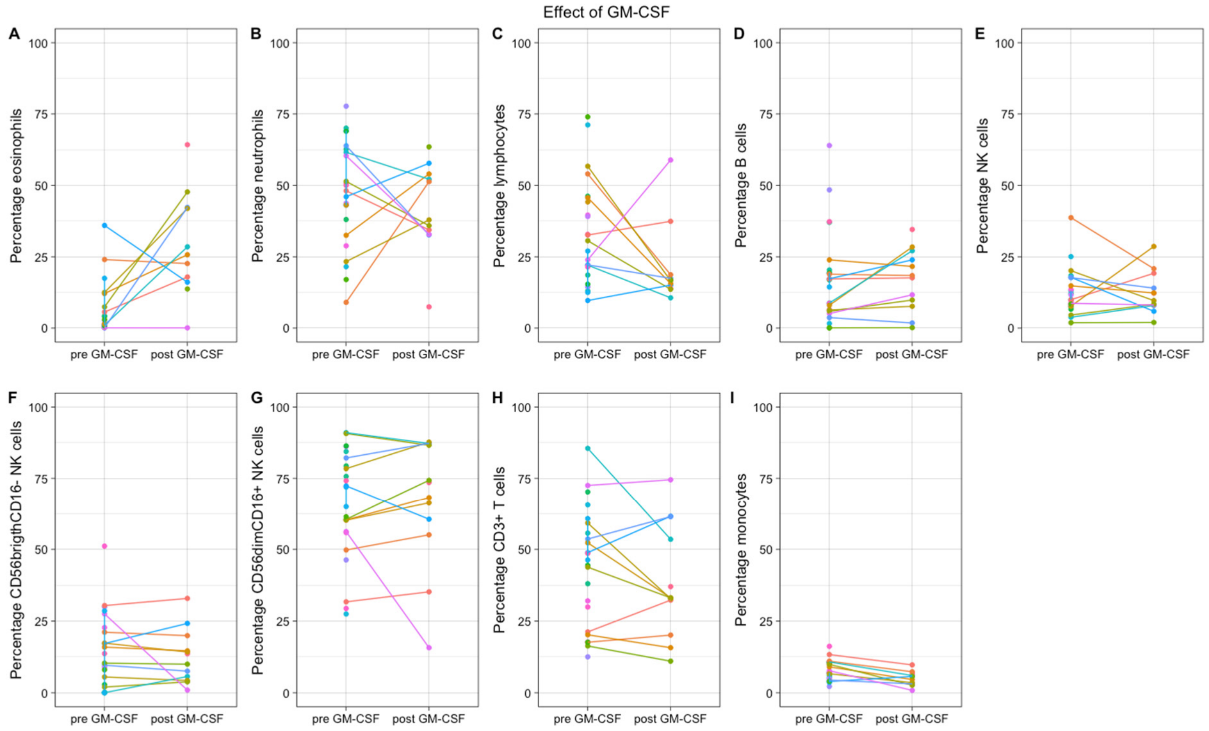

Figure S3. Percentages of cell types based on trucount data before and after GM-CSF containing immunotherapy cycles.

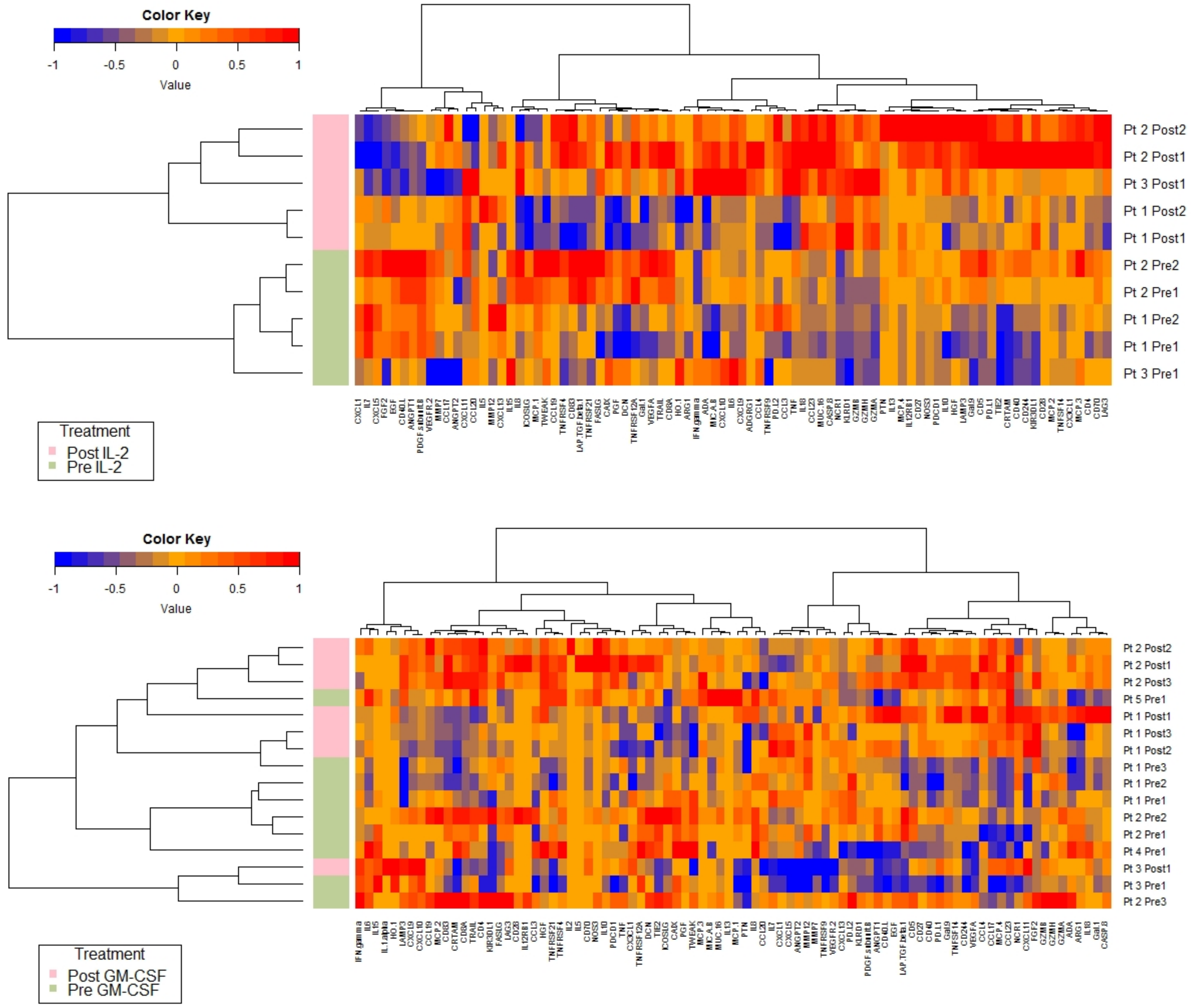

Figure S4. Clustering of immune-oncology related plasma protein concentrations of patients pre- and post-IL2 and GM-CSF containing immunotherapy cycles. Unsupervised clustering of 92 immuno-oncology related biomarkers. (**A**) Complete separation of protein profiles pre- and post IL-2 containing IT-cycles. From three patients, samples were paired before (day 1 IT-cycle 2 or 4) and after (day 15 IT-cycle 2 or 4) IL-2 containing IT. In total, 5 paired samples are shown, as two patients were monitored during both IT-cycles. (**B**) Partial separation of protein profiles pre- and post GM-CSF containing IT-cycles. From three patients, samples were paired before (day 1 IT-cycle 1,3 or 5) and after (day 15 IT-cycle 1,3 or 5) GM-CSF containing IT. In total, 7 paired samples are shown, as two patients were monitored during all three IT-cycles. 2 single measurements from patients pre GM-CSF were included, resulting in a total of 9 patients pre- and 7 post GM-CSF. Protein profiles of patient 3 (GM-CSF cycle 1) cluster together, indicating the absence of major differences in protein profiles in this patient upon GM-CSF containing IT. In addition, Protein profiles of patient 5 (unpaired, pre-GM-CSF) did cluster together with protein profiles of other patients post-GM-CSF.
